# Usefulness of C-reactive protein and serum amyloid A in early detection of postoperative infectious complications to tibial plateau leveling osteotomy in dogs

**DOI:** 10.1186/s13028-018-0385-5

**Published:** 2018-05-21

**Authors:** Karin Löfqvist, Mads Kjelgaard-Hansen, Michelle Brønniche Møller Nielsen

**Affiliations:** 1 AniCura Animal Hospital in Hässleholm, Stjärnfallsvägen 9, 281 43 Hässleholm, Sweden; 2 Department of Veterinary Clinical Sciences, Faculty of Health and Medical Sciences, University of Copenhagen, Dyrlaegevej 16, 1870 Frederiksberg C, Denmark

**Keywords:** C-reactive protein, Dog, Infectious complication, Orthopedic surgery, Serum amyloid A, Tibial plateau leveling osteotomy (TPLO)

## Abstract

**Background:**

Cranial cruciate ligament rupture is a prevalent injury in dogs, and tibial plateau leveling osteotomy (TPLO) is one of the preferred surgical techniques. Surgical site infection is a possible complication following TPLO and measurement of serum acute phase proteins is suggested to be a way to early recognize and distinguish postoperative infectious complications from normal postoperative inflammatory conditions. In this study we investigate the changes in concentrations of the systemic inflammatory markers C-reactive protein (CRP) and serum amyloid A (SAA) following tibial plateau leveling osteotomy (TPLO) in dogs and evaluate if deviations from the changes expectedly induced by the surgical procedure are useful in early detection of post-surgical infections. Dogs with cranial cruciate ligament injuries treated by TPLO at the Region Animal Hospital of Helsingborg during 2012 were included. Dogs with concurrent diseases, other orthopedic problems, or noninfectious post-surgical complications were excluded. Serial measurements of CRP and SAA concentrations were made. Changes in concentrations were visualized graphically and the discriminative capacity to detect infectious post-surgical complications was tested at different time points.

**Results:**

A characteristic pattern of changes in concentrations of CRP and SAA were observed following TPLO with a significant increase 24 h post-surgery in all dogs and baseline-concentrations re-established at day 12. In dogs that developed post-surgical infections, a deviation in form of significantly higher concentrations of CRP and SAA were observed at day 6, compared to un-complicated cases. High-discriminative clinical decision limits of CRP (43.9 mg/L) and SAA (63.8 mg/L) could be established for differentiation of dogs with and without clinical signs of infectious complications at day 6 post-operatively, applicable to reliably rule out presence of infectious complications due to very high sensitivity (no false negatives).

**Conclusions:**

The CRP and SAA levels in dogs with clinical signs of post-surgical infectious complication deviated from the typical levels expected at day 6 after surgery, and clinical decision limits to reliably rule out presence of infectious complications was suggested.

## Background

Cranial cruciate ligament (CrCl) rupture is a prevalent injury in dogs and of great economic importance in canine veterinary practice [1]. Tibial plateau leveling osteotomy (TPLO) is one of the preferred surgical techniques to achieve functional stifle joint stability following CrCl injury, and the surgical method has been validated in several studies [2, 3]. Different types of postoperative complications have, however, been reported following TPLO surgery, including infectious complications as well as non-infectious complications like meniscal tears, tibial crest fractures and the formation of seromas [4–9]. Complication rates between 10 and 34% have been reported [4, 6–9], including complications of infectious origin, which are considered to be higher in TPLOs (0.8–14.3%) [6–8, 10–12] compared to other elective orthopedic procedures (1.5–2.6%) [11, 13, 14]. The risk of infectious complication is further increased in carriers of methicillin-resistant *Staphylococcus pseudintermedius* [15].

Complications usually pose an animal welfare implication and the earlier the complication can be identified and treated, the less the animals’ suffering, and the better the expected outcome will be [5, 9]. Complications are also associated with increased cost [16]. The need for further surgical intervention is more frequent if complications are recognized late in the postoperative period [12], whereas a less costly treatment is often sufficient in case of complications detected during the early postoperative period. Thus an objective mean for early detection of complications should be useful from both an animal welfare and an economical point of view.

Acute phase proteins (APPs) can be used as markers of systemic inflammation in dogs [17]. C-reactive protein (CRP) and serum amyloid A (SAA) are useful APPs in dogs, and automated assays have been validated for diagnostic measurements of both proteins [18, 19], making them applicable for routine use [20]. Serum concentrations of canine CRP and SAA will increase significantly in response to various stimuli causing systemic inflammation, and because of the short half-lives of these proteins, decreasing concentrations will quickly be observed corresponding to decreasing inflammatory activity [17, 20]. As a result, characteristic patterns of changes in serum CRP and SAA concentrations are observed following standardized aseptic surgery as ovariohysterectomy [21], and deviation from such a characteristic response has proven to hold potential in the detection of inflammatory post-surgical complications [22]. Even though further documentation is necessary in order to increase the level of evidence [23], routine serial measurements of serum CRP and SAA can also be expected to hold a potential in the detection of inflammatory complications following other kinds of standardized surgery in dogs [24]. Orthopedic patients have, however, only occasionally been included in studies also including canine APPs [25]. Therefore, further studies are needed in order to investigate the diagnostic potential of APPs in dogs with CrCl rupture or other orthopedic conditions, and the present study is one step towards further knowledge in this field.

The aim of the present study was to investigate if postoperative measurements of CRP and SAA following TPLO surgery in dogs can be useful in the detection of early infectious complications and thereby provide an assisting tool for the clinician to differentiate infectious cases from normal postoperative swelling and bruising.

## Methods

The study was conducted as a prospective, longitudinal, observational study including client owned dogs presented for surgical treatment of CrCl injury by the TPLO procedure at the Region Animal Hospital of Helsingborg from January to December 2012.

### Pre-surgical work-up

Within 24 h prior to the surgical procedure, the dogs underwent thorough clinical examination including assessment of the general condition, body temperature measurement, visual and audible examination of circulation and respiration, visual and palpatory examination of skin, mucous membranes, oral cavity, genitalia, the locomotor apparatus and palpatory examination of the abdominal organs. Standard hematological and biochemical profiles were analyzed at the local clinical pathological laboratory to ensure that only clinically healthy dogs were included in the study. With clinically healthy, dogs with good general condition and no detectable signs of disease that could stem from an infectious or systemic inflammatory condition, were intended. The laboratory analyses included hematological differential counts of red and white blood cells and platelets, microscopic assessment of leukocyte morphology, and analysis of the biochemical parameters natrium, potassium, calcium, alkalic phosphatase, alaninaminotransferas, creatinine, urea, cholesterol, protein, albumin and globulin. The laboratory analysis also included measurements of serum CRP using a point of care magnetic permeability immunoassay (Life-Assays, Sweden).

Inclusion criteria consisted of dogs with good general condition and no signs of infectious or systemic inflammatory disease that were presented to the Regional Animal Hospital in Helsingborg during 2012 for TPLO surgery due to a cranial cruciate ligament injury and where the owners had given their written permission to participate in the study. Dogs were consequently only included in the study if no substantial deviations in clinical or clinicopathological examinations compatible with pathological activities could be demonstrated. Close clinical attention was paid to rule out other reasons for limping on the affected leg than the cruciate ligament injury. Thorough palpatory examination of the leg was performed in order to identify any pathological changes but the injured knee. Dogs with any signs of other orthopedic problems were consequently excluded from the study.

The exclusion criteria consisted of signs of infectious or systemic inflammatory disease, raised CRP preoperatively, marked biochemical or hematological abnormalities, evidence of significant pathological findings in the general clinical examination or any other reason for limping on the affected leg than the cruciate disease.

### Surgery, anesthesia and analgesia

The dogs were premedicated with morphine (0.3 mg/kg intramuscularly [IM]), carprofen (0.08 mg/kg IM) and diazepam (0.2 mg/kg intravenously [IV]). Anaesthesia was induced with propofol IV and maintained with isoflurane inhalation in oxygen/air. Epidural morphine (0.1 mg/kg) was given in the lumbosacral space immediately preoperatively. No prophylactic antibiotics were given. An intra-articular deposition of bupivacaine (0.5 mg/kg) was given after suturing the joint capsule intra-operatively.

All dogs underwent TPLO *lege artis* according to the same procedure introduced by Slocum and Slocum in 1993 [26] and performed by the same experienced small animal surgeon. Open joint assisted arthroscopy was performed through a craniomedial parapatellar mini-arthrotomy in all dogs [27].

### Post-surgical work-up

#### Pilot study to explore optimal timing for sampling

To explore the optimal timing of sampling for detection of changes in serum CRP and SAA concentrations following TPLO in dogs, a pilot study was conducted including the 5 first operated dogs. Thorough clinical examinations as described below pre-surgical work-up, were performed, and follow-up serum samples were obtained before and 24 h after surgery, and sequentially at 3, 6, 9, 12, 15, 18, and 21 days after surgery. All serum samples were frozen at − 20 °C for a maximum of 9 months, and were subsequently transported on wet-ice to the Central Laboratory of the Department of Veterinary Clinical Sciences, University of Copenhagen for duplicate CRP and SAA analyses within a few hours from the departure from Helsingborg. Serum CRP was measured with a commercially available human turbidimetric immunoassay (High linearity CRP, Randox, Crumlin, County Antrim, UK) using species-specific calibration material [28]. Serum amyloid A was measured by an automated latex agglutination turbidimetric immunoassay (SAA-1, Eiken Chemical Company, Japan) using heterologous human SAA for calibration [18]. Both parameters were analyzed using an automated clinical chemical analyzer (Advia 1800, Siemens, Munich, Germany), and both assays have been validated for diagnostic use in dogs [18, 28, 29]. Changes in serum CRP and SAA concentrations were visualized graphically, which indicated that in uncomplicated cases, serum CRP and SAA concentrations peaked 1 day post-surgery and then gradually declined to reach the referral ranges for healthy dogs 6 days postoperatively, and all dogs reached baseline levels 12 days postoperatively if no further complication developed afterwards.

#### Remaining study

The 5 dogs included in the pilot study were also included in the remaining study. Serum was obtained pre-surgically from the remaining dogs included in the study and at the 3 time-points described above; 1, 6 and 12 days post-surgery, for follow-up sampling and clinical assessments. All samples were analyzed on the same analytical batch in order to avoid between-run imprecision. Consequently, samples included in the pilot study as well as the remaining study were analyzed twice.

A thorough physical examination was conducted on each dog in order to identify signs of postoperative infectious complications. The same small animal surgeon performed all clinical assessments according to a standardized protocol exclusively designed to detect infectious complications. During the clinical examination the general condition was assessed, as well as the body temperature, the swelling of the wound, the swelling of the entire leg, the redness of the wound, the drainage from the wound, the pain on palpation of the knee, the pain on palpation of the entire leg and the range of motion of the knee joint. The degree of lameness was assessed, the circumference of the thigh was measured on several fixed points and the joint angle was measured with goniometry. Infectious complications were defined as reduced general clinical condition, that is, some degree of depression, increasing lameness later than 24 h after surgery, moderate or severe swelling of the surgical wound, secretion from the surgical wound, increasing degrees of swelling of the operated leg later than 24 h after surgery, moderate or severe redness of the wound arisen later than 24 h after surgery, and a moderate or severe pain on palpation of the wound arisen later than 24 h after surgery. A post-operative infection was defined as a condition where 4 or more of the above signs were present simultaneously. A post-operative infectious complication was also considered if the surgical area was affected with purulent drainage, a fistula or an abscess, or if an infection was detected by bacterial culture. All clinical assessments were made blinded to the APP-results.

Dogs with noninfectious complications following TPLO were excluded from the study. Non-infectious complications were defined as abnormal conditions that do not meet the above set criteria for infectious complications. In these patients no antibacterial treatment was needed for fully recovery, but the patient still suffered from a deterioration from recovery, for example as a result of meniscal tears, tibial crest fractures and formations of seromas.

### Statistics

Concentrations of serum CRP and SAA in dogs with and without post-surgical infectious complications were visualized graphically using Box- and Whisker plots, and median and ranges were calculated in order to illustrate any apparent differences in concentrations at each of the 4 time-points. Differences in concentrations at post-surgical time points compared to baseline concentrations were tested by parametric paired t-tests. Differences between serum CRP and SAA concentrations in dogs with and without infectious complications were tested for significance using Mann–Whitney tests. Significance level was set to P < 0.05. For any time-point demonstrating significant differences, the diagnostic performance of serum CRP and SAA concentrations to distinguish infectious complications to TPLO from cases without infectious problems was further assessed by assessment of the area under the curves (AUC) obtained by receiver operating characteristic (ROC) analyses. The parameters were considered to have diagnostic potential for the detection of post-surgical infectious complications if the AUC was found to be above 0.5 (significance level P < 0.05). Further, the ROC analysis was used to identify relevant clinical decision limits, defined as the concentration at the maximum differential positive rate [maxDPR, where DPR = sensitivity − (1 − specificity)].

## Results

Thirty dogs were presented for TPLO at the Region Animal Hospital of Helsingborg during the study period. Eleven dogs were excluded from the study. Of these, 5 dogs showed signs of other orthopedic problems. The owners of another 5 dogs rejected participation because of the frequent revisits. One dog had a mild, transient lameness in the early post-operative period, which quickly resolved without any medical treatment. Because of the quick, spontaneous recovery an infectious complication seemed unlikely. On the other hand, because of the unexpected, transient symptoms, the dog could reliably not be classified together with the dogs without complications. Consequently, this dog was excluded from the study. Nineteen dogs were included in the study. Three of the included dogs developed clinical and microbiological signs of infectious complications post-TPLO and one dog showed obvious signs of infection clinically and on synovial fluid cytology. The 4 dogs were all treated with antibiotics. Typical signs within these dogs were increased lameness and swelling of the operated leg, increased redness and pain on palpation of the surgical site, and sometimes drainage from the wound. Of the 4 dogs affected by a post-operative infectious complication, 3 had secretions from the surgical wound and were cultured, 3 underwent arthrocentesis, and 1 was radiographed in order to confirm the diagnosis by excluding other causes for the clinical signs of illness. Fifteen dogs had a completely uneventful postoperative period with no signs of infectious complications.

**Fig. 1:**
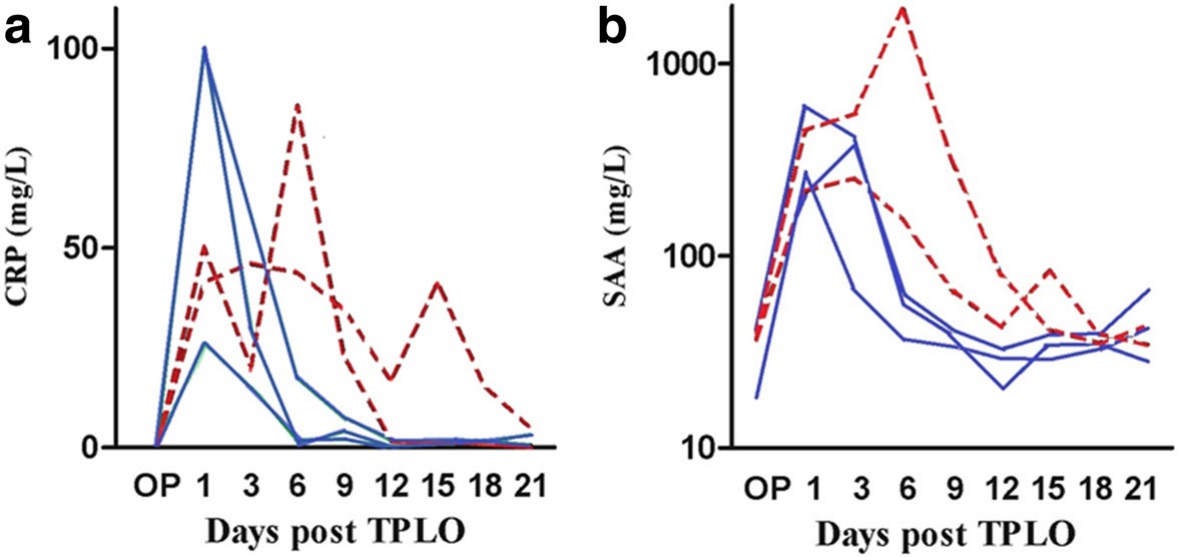
Pre- and postsurgical serum concentrations of C-reactive protein (CRP, **a**) and serum amyloid A (SAA, **b**) measured in 5 dogs with cranial cruciate ligament rupture treated surgically by tibial plateau leveling osteotomy (TPLO). Note the significant increase of serum CRP and SAA concentrations during the first 24 h post-surgery and the tendency to reach baseline levels at day 12 in uncomplicated cases. Note the tendency of higher serum concentrations of CRP and SAA at day 6 in dogs which developed soft tissue infections post operatively (broken lines), while no such increases were observed in dogs with no signs of postsurgical infections (solid lines)

As illustrated in Fig. 1, serum concentrations of SAA and CRP were measured before and 1, 3, 6, 9, 12, 15, 18, and 21 days after TPLO in the 5 dogs included in the first part of the study. Two of these dogs developed infectious complications post-TPLO. Increasing serum concentrations of CRP and SAA were observed during the first 24 h post-TPLO in all dogs. While both proteins reached pre-surgical concentrations 6 days post-TPLO in uncomplicated cases, serum concentrations were still increased after 6 days in dogs that developed infectious complications. At day 12, pre-surgical concentrations seemed to be reached in all dogs if further infectious complications did not develop afterwards (Fig. 1).

**Fig. 2:**
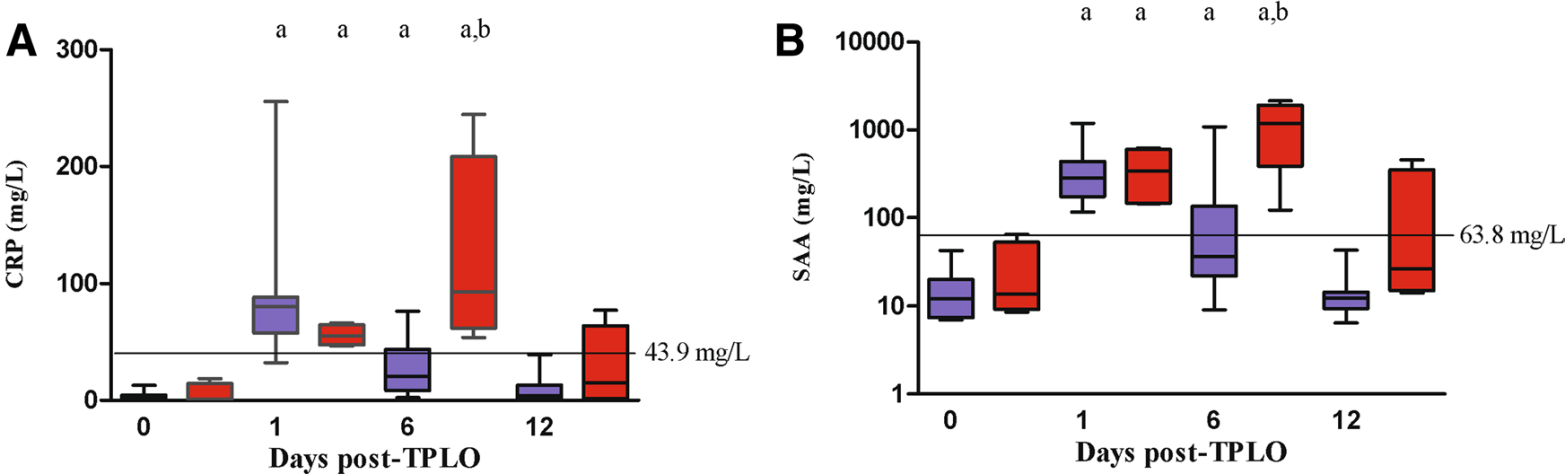
C-reactive protein (CRP, **A**) and serum amyloid A (SAA, **B**) concentrations in canine serum before and 1, 6 and 12 days following tibial plateau leveling osteotomy (TPLO). Dogs, with (red) and without (blue) infectious complications to surgery, were included in the study. Statistically significant higher serum concentrations were observed day 1 and 6 compared to baseline concentrations at day 0 in dogs with and without infectious post-surgery complications (a). On day 6, statistically significant higher serum concentrations were observed in dogs with infectious complications to surgery, compared to dogs with no complications (b). Baseline concentrations were reached again on day 12. Significance level was set to P < 0.05. The optimal clinical decision limits of serum SAA and CRP concentrations to discriminate dogs with and without infectious complications (horizontal lines) were established by receiver operating characteristic curve analyses

**Fig. 3:**
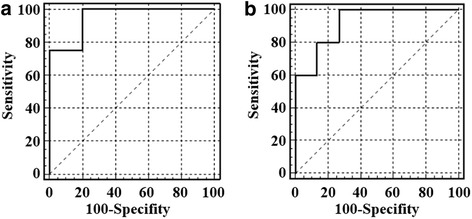
Receiver operating characteristic (ROC) curves (solid lines) illustrating the diagnostic sensitivity and specificity of C-reactive protein (CRP, **a**) and serum amyloid A (SAA, **b**) in the detection of infectious complications following tibial plateau leveling osteotomy in dogs. Note that the area under both ROC curves are significantly higher than 0.5 (dashed lines)

In all 19 dogs included in the study, including the dogs in the pilot-study, higher serum concentrations of CRP and SAA were observed 1 and 6 days post-surgery compared to pre-surgical concentrations (Fig. 2). Pre-surgical concentrations were approached 12 days post-surgery in most dogs, confirming the tendencies observed in the pilot study (Fig. 1). Significantly higher serum concentrations of CRP and SAA were observed 6 days post-surgery in dogs that developed infectious complications [median (range), CRP 92.9 mg/L (53.8; 244.6 mg/L) and SAA 1184 mg/L (122.8; 2184 mg/L)] compared to dogs with no clinical signs of infectious complications [CRP 20.6 mg/L (2.3; 76.4) and SAA 36.7 mg/L (9.1; 1088 mg/L)]. At the other time-points, no significant differences could be demonstrated between dogs with and without infectious complications (Fig. 2). The diagnostic potential for serum CRP and SAA in the detection of post-surgical infectious complications was further confirmed by the ROC-curves (Fig. 3), showing AUCs significantly higher than 0.5 [AUC (95% CIs) CRP 0.95 (0.74; 0.99), SAA 0.92 (0.71; 0.99)]. This means that a randomly selected dog with infectious complications will have higher CRP and SAA concentrations than a randomly selected dog with no complications in 95 and 92% of the time, respectively. As illustrated in Fig. 2, clinical decision limits for CRP (43.9 mg/L) and SAA (63.8 mg/L) could be established corresponding to the maximal differential positive rates. Special attention should be directed to that the decision limits for both CRP and SAA were well below the respective concentrations observed for dogs with signs of infectious complications, thus showing perfect sensitivity and no false-negatives in the studied population.

## Discussion

This is the first study of APPs following TPLO in dogs, and furthermore to suggest their use to reliably support ruling-out presence of post-surgical infectious complications at an early time point (day 6). In surgical patients, APPs have been most extensively studied following elective ovariohysterectomy [21, 24, 30, 31] or orchidectomy [24, 32] of clinically healthy dogs, but significant changes in the concentrations of APPs also have been demonstrated following other types of surgery there amongst several orthopedic procedures including wedge resection trochleoplasty with tibial crest transposition for surgical treatment of patella luxation and different techniques for surgical treatment of CrCl rupture (modified de Angelis lateral fabella suture and over the top fascial graft) [25]. Different outcomes of the postoperative fluctuations in serum CRP and SAA would be to expect regarding clean procedures, like TPLO, compared to clean contaminated or contaminated procedures. In the clean procedure the normal postoperative fluctuations in APPs are due to the inflammation caused by surgery itself. In the clean contaminated or the contaminated surgery the postoperative fluctuations in APPs are not only influenced by surgery, but also from the inflammation induced by a variable amount of microorganisms that invaded the surgical wound in connection with, or after surgery.

TPLO is a highly invasive procedure and some postoperative swelling is common, often making it difficult to decide whether there are clinical signs of infectious complications or not [8–10]. Measuring serum concentrations of CRP and SAA may help the clinician to differentiate doubtful cases of normal postoperative variation from early postoperative infection without the need of more invasive, expensive, or time-consuming tests. However, measurement of APPs cannot replace a thorough clinical examination. As other unspecific markers of systemic inflammation, serum concentrations of CRP and SAA can be expected to increase in dogs with systemic inflammation of any cause [17, 20, 33]. However, with a thorough clinical examination exclusively identifying abnormalities related to the surgical site, APPs can be used to further evaluate these abnormalities for possible systemic inflammatory involvement. Further, even though serial repeated phlebotomy sampling will be needed, these will not in itself cause increases in major APPs [34].

Only dogs with no or infectious complications to TPLO were included in the present study. However, increased serum concentrations of CRP and SAA may occur as a result of all complications involving systemic inflammation. Further studies are needed in order to investigate whether serum CRP and SAA concentrations are useful in order to differentiate dogs with infectious complications post-TPLO from dogs with other complications involving inflammation, e.g. the expected local inflammatory response following meniscal injuries or tibial crest fractures. On the other hand, various grades of infectious complications are expected in a general clinical setting. Thus, patients with various numbers and combinations of symptoms of infectious complications can be expected in the clinic—not necessarily fulfilling the set criteria for postoperative infectious complication used in the present study. Further studies are needed in order to investigate the concentrations of CRP and SAA in patients with noninfectious and various degrees of infectious complications, respectively, and such studies are necessary before CRP and SAA can be implemented as reliable diagnostic parameters routinely used to assess the post-operative period following TPLO in dogs. However, the present study is an important first step toward this goal, as the parameters are demonstrated to be useful to rule out infectious complications, thus demonstrating a good overlap performance in a population of dogs with clear infections versus dogs with no signs of infection.

In the present study, clinical decision limits of serum concentrations of CRP and SAA were established in order to differentiate dogs with infectious complications from dogs with no signs of post-surgical complications. The term clinical decision limit refers to certain levels of serum CRP and SAA where some kind of medical action is recommended to provide the patient with proper care. ROC-curves have previously been used to establish clinical decision limits of canine serum SAA and CRP differentiating dogs with and without systemic inflammation in our laboratory [20, 33]. For SAA, it is, however, important to note that such limits should be established locally because of the heterologous nature of the calibration material [35]. The decision limits established in the present study are higher than the decision limits for detection of systemic inflammation in the previous studies [20, 33]. This is because the decision limits established 6 days following TPLO also account for the grade of systemic inflammation, which can be expected as a result of the invasiveness of surgery itself, compared to the results obtained in previous studies comparing dogs with and without systemic inflammation of any cause.

In the present study, the rate of over all complications was within earlier reported rates [4, 6–9]. Reservation should be made with reference to the limited postoperative period the study compromise. However, infectious complication rates were considerably higher than previously reported (21% compared to 0.8–14.3%) [6–8, 10, 11]. A possible explanation could be that no prophylactic antibiotics were used in the present study despite documented recommendations [36], or due to other complicating factors like presence of MRSP carriers amongst the included patients, reported to increase the risk [15], however which was not within the scope of the current study to investigate. According to the authors’ personal experience, infections are one of the complications most commonly seen in the early postoperative period, which the study covers. The main challenge in these cases is to distinguish self-relieving postoperative swellings, which may well cause a noticeable discomfort, from the postoperative infections. The clinical application of the results of this study, is that the clinician can, with the support of single or repeated measurements of serum CRP and SAA, together with careful clinical investigation, more easily determine whether an infectious complication that causes further investigations, exists or not. A single value of serum CRP above 43.9 mg/L and/or serum SAA above 63.8 mg/L 6 days or more postoperatively together with deteriorated clinical improvement, or repeated serum samples with increased CRP or SAA concentrations later than 24 h postoperatively together with deteriorated clinical improvement, suggest that an infectious complication could be possible and should be further investigated.

## Conclusions

Characteristic patterns of changes in serum CRP and SAA concentrations were demonstrated following TPLO in dogs, and if no deviations from this characteristic response are observed 6 days post-surgery, post-surgical infectious complications can seemingly be ruled out. The use of APPs postoperatively will, thus, provide the clinician with an objective marker to support the clinical assessments in order to detect infectious complications to TPLO.

## Abbreviations

APP: acute phase protein
AUC: area under the curve
CrCl: cranial cruciate ligament
CRP: C reactive protein
DPR: differential positive rate
IM: intramuscular
IV: intravenously
ROC: receiver operating characteristic
SAA: serum amyloid A
TPLO: tibial plateau leveling osteotomy
